# Measuring Annual Variation in Reproductive Output Reveals a Key Role of Maternal Body Condition in Determining the Size of Eggs in Snakes

**DOI:** 10.3390/ani12121494

**Published:** 2022-06-08

**Authors:** Kun Guo, Xiang-Mo Li, Yan-Qing Wu, Yan-Fu Qu, Xiang Ji

**Affiliations:** 1 Zhejiang Provincial Key Laboratory for Water Environment and Marine Biological Resources Protection, College of Life and Environmental Sciences, Wenzhou University, Wenzhou 325035, China; guokun8808@wzu.edu.cn; 2 Jiangsu Key Laboratory for Biodiversity and Biotechnology, College of Life Sciences, Nanjing Normal University, Nanjing 210042, China; lixiangmo@njnu.edu.cn; 3 Key Laboratory of Biosafety, Nanjing Institute of Environmental Sciences, Ministry of Ecology and Environment, Nanjing 210023, China; wyq0710308@126.com

**Keywords:** clutch size, clutch mass, Colubridae, egg size, female reproduction, life history variability

## Abstract

**Simple Summary:**

We measured female reproductive traits of a colubrid snake (*Lycodon rufozonatus*) from Zhejiang, East China in four years (1999, 2010, 2011, and 2014). After removing the influence of female size, postpartum body mass was greater in 2010 than in 2014, clutch mass was greater in 2010 than in 2011 and 2014, and egg size was greater in 2010 than in the other three years. Egg size was positively related to postpartum body condition in each year. Females laid larger eggs in 2010 than in other three years after removing the influence of maternal size and body condition. Our study does not follow the prediction that reproductive females with different amounts of resources to invest should give priority to adjusting the number rather than size of offspring.

**Abstract:**

Long-term studies are especially suited for disentangling the effects of extrinsic and intrinsic factors on both total reproductive investment and reproductive allocation in offspring number versus offspring size. Female reproductive traits of the red-banded wolf snake (*Lycodon rufozonatus*) from Zhejiang, East China were studied in four years between 1999 and 2014. Egg-laying dates overall extended from late June to late July, and varied among years. Postpartum body mass, clutch size, clutch mass, and egg size were positively related to female size (snout vent length, SVL) in each year. Postpartum body mass, clutch mass, and egg size differed among years after accounting for female SVL, whereas clutch size did not. Setting female SVL at the same level, postpartum body mass was greater in 2010 than in 2014, clutch mass was greater in 2010 than in 2011 and 2014, and egg size was greater in 2010 than in the other three years. Females did not trade off egg size against number. Egg size was positively related to postpartum body condition in each year. Females laid larger eggs in 2010 than in other three years after removing the influence of maternal body condition. Our study provides evidence for the traditional view that reproductive output is highly linked to maternal body size in snakes, but not following Smith and Fretwell’s (1974) classic prediction that females with different amounts of resources to invest in reproduction should give priority to adjusting the number rather than size of their offspring. Maternal body size and condition both are important sources of variation in egg size, but factors other than these two variables may also affect the size of eggs produced by female *L. rufozonatus*.

## 1. Introduction

The number and size of offspring produced by a female are of central interest in life history studies because they not only determine reproductive output but also influence microevolutionary fitness [1,2,3,4]. The classic theory of resource partitioning among individual offspring predicts that variation in resources available to females for reproduction should be reflected as changes in the number rather than the size of offspring [5]. Following this prediction, females with different amounts of resources to invest in reproduction should give priority to adjusting the number rather than size of their offspring, such that offspring size should be relatively invariant, or vary little among individuals of the same population. Contrary to this prediction, increasingly more empirical evidence from diverse animal taxa indicates that females can adjust offspring size by assessing the environment their offspring will encounter based on their own experience [6,7,8,9,10], or offspring size can vary in response to changes in total reproductive investment, fecundity, and maternal size and/or body condition [11,12,13,14].

Studies by experimentally manipulating clutch or litter size of squamate reptiles show that offspring (egg or neonate) size (mass) varies with offspring number in all species studied thus far [15,16,17,18,19,20], with only one exception of *Takydromus septentrionalis* [21], a lacertid lizard where females can produce up to nine clutches per breeding season [22]. Unlike females of other species reproducing less frequently, female *T. septentrionalis* tend to divert a fixed fraction of resources to individual offspring in a reproductive episode and channel current surplus resources into the next clutch, thereby maximizing the number of offspring produced per season [21,22,23,24]. Taken together, previous studies consistently suggest that life history trade-offs are the major constraint that conditions the resolution of resource allocation among competing demands and determines how resources allocated to reproduction are divided among individual offspring and between current and future reproductive events.

Long-term studies are especially suited for disentangling the effects of past and present, and extrinsic and intrinsic factors on both total reproductive investment and reproductive allocation in clutch or litter size versus offspring size. Squamate reptiles are well-suited for studies on offspring size variation because they often lack parental care and, in the vast majority of these animals, reproductive investment per offspring is completed at ovulation (for oviparous and viviparous species with lecithotrophy) or parturition (for viviparous species with various degrees of placentotrophy) [25]. However, as in other animal taxa, life history trade-offs in squamate reptiles are difficult to detect without measuring the traits in question over time or under contrasting environmental conditions [11,26,27]. Here, we reported data on female reproductive traits collected in four years (1999, 2010, 2011, and 2014) for an oviparous colubrid the red-banded wolf snake *Lycodon* (formerly *Dinodon*) *rufozonatus* (Colubridae) from Zhejiang Province, East China, paying particular attention to the traits that vary among years and factors that have a key role in determining egg size.

## 2. Materials and Methods

### 2.1. Study Species

*Lycodon rufozonatus* is a medium-sized (up to 1120 mm snout vent length, SVL) oviparous colubrid snake that can be found in most provinces including Taiwan of China, absent only from Inner Mongolia, Qinghai, Xinjiang, and Tibet; it also occurs in Korea, easternmost Russia, northern Laos and Vietnam, and the Ryukyu Archipelago [28]. The snake uses diverse habitats in the hilly and lowland countryside, displaying male-biased sexual size dimorphism [28]. From previous life history studies of *L. rufozonatus,* we know the following. First, females larger than 690 mm SVL lay a single clutch of 5–20 eggs per egg-laying season stretching from late June to late July [29]. Second, eggs can be successfully incubated at temperatures ranging from 24–30 °C, with mean incubation lengths varying from ~46 d at 30 °C and ~76 d at 24 °C [30,31]. Third, clutch size is determined about one month after winter dormancy, soon after the initiation of vitellogenesis in late April; larger females lay larger and heavier clutches than smaller ones do [29].

### 2.2. Animal Collection and Care

**Figure 1:**
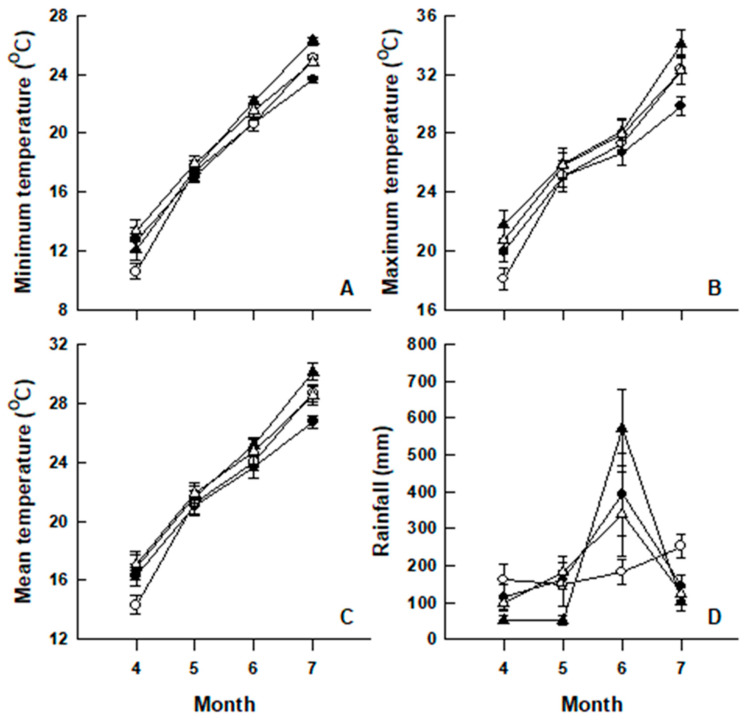
Mean values (±SE) for monthly minimum (**A**), maximum (**B**) and mean (**C**) air temperatures and rainfall (**D**) over a four-month period from April to July in 1999 (●), 2010 (○), 2011 (▲), and 2014 (△) (http://www.cma.gov.cn; accessed on 28 February 2022).

We collected 136 gravid females in mid-June of 1999 (*N* = 35), 2010 (*N* = 34), 2011 (*N* = 21), and 2014 (*N* = 48) from various localities (28°35′–30°16′ N, 119°15′–122°10′ E) in Zhejiang Province, East China. Two-way ANOVAs, with month and years as the fixed factors on climatic data (provincial mean values for daily minimal temperature, maximal temperature, mean temperature, and rainfall) downloaded from the website of China Meteorological Administration (http://www.cma.gov.cn; accessed on 28 February 2022) for the period (from April to July) soon after hibernation but before the end of the breeding season, revealed the following. First, monthly minimum, maximum, and mean temperatures varied among the four months (*p* < 0.001 in all cases) and among the four years (*p* < 0.01 in all cases). Second, monthly rainfall varied among months (*F*_3,32_ = 19.25, *p* < 0.001) but not among years (*F*_3,32_ = 0.09, *p* = 0.968). Third, the month × year interaction was a significant source of variation in monthly minimum temperature and rainfall (*p* < 0.05 in both cases) but not in the other two climatic variables (*p* > 0.05 in both cases) (Figure 1).

Females collected in each year were brought to our laboratory, where two or three individuals were housed together in each 500 × 450 × 350 mm (length × width × height) wire cage. We placed cages in a room where temperatures varied from 24–30 °C optimal for egg incubation [30]. We provided cricket frogs (*Fejervarya limnocharis*) and water enriched with multivitamins and minerals ad libitum. We collected and weighed eggs within 6 h post-laying and used clutch mean egg mass as a proxy for egg size throughout the text. Egg-laying date, SVL, and body mass were taken for each postpartum female. Eggs were incubated under multiple thermal conditions, and data have been [30,31] and will be reported elsewhere. We released females collected in each year at their point of capture in early August soon after the breeding season.

### 2.3. Data Analyses

Seven females (three in 1999, two in 2010, and two in 2014) that laid abnormal eggs with condensed yolk were excluded from analyses. Clutch mass was the total mass of eggs in a clutch, and relative clutch mass (RCM) was calculated by dividing clutch mass by postpartum body mass [32]. Maternal body condition (mass relative to SVL) was represented by residual score from the regression of log_e_ (postpartum body mass) against log_e_ (SVL) [26]. Within-clutch egg size variability was analyzed using the coefficient of variation [CV = 100 × (standard deviation/mean)], as was within-population variability in clutch size, clutch mass, and egg size.

All statistical analyses were performed with Statistica 8.0 (Tulsa, OK, USA). Before parametric analyses, data were tested for normality using the Kolmogorov–Smirnov test and for homogeneity of variances using Bartlett’s test. Data were log_e_ transformed when necessary to meet the assumptions for parametric analysis. We used linear regression analysis, one-way ANOVA, one-way ANCOVA, or partial correlation analysis to analyze the data. Slope homogeneity was checked prior to ANCOVAs. Tukey’s post hoc test was performed on the traits that differed among years. Throughout this paper, values are presented as mean ± standard error (SE), and the significance level is set at *p* = 0.05.

## 3. Results

**Figure 2:**
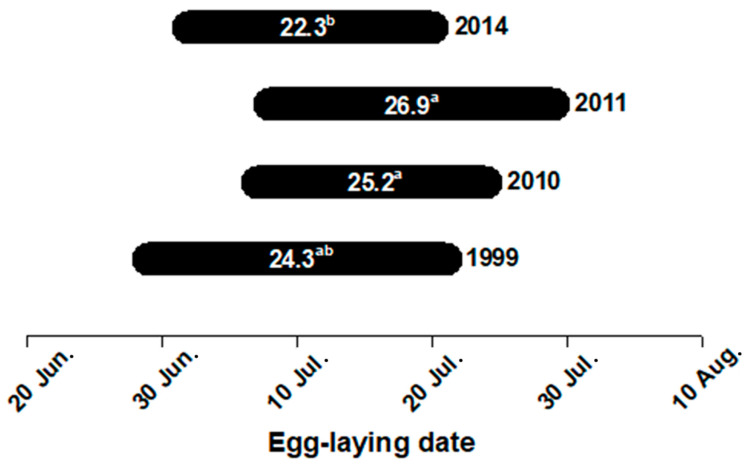
Egg-laying dates in four different years. Numbers on the horizontal bars indicate the mean number of days starting from 20 June. Means with different superscripts differ significantly (Tukey’s *post hoc* test, α = 0.05, a > b).

Reproductive female sizes ranged from 625–1065 mm SVL, with an overall mean SVL of 818.5 mm; mean female SVL did not differ among years (Table 1). Egg-laying dates overall extended from late June to late July and varied among years (one-way ANOVA: *F*_3,127_ = 4.89, *p* < 0.01), with females laying eggs significantly earlier in 2014 than in 2010 (advanced by a mean of ~3 days) and 2011 (advanced by a mean of ~5 days) (Figure 2).

**Figure 3:**
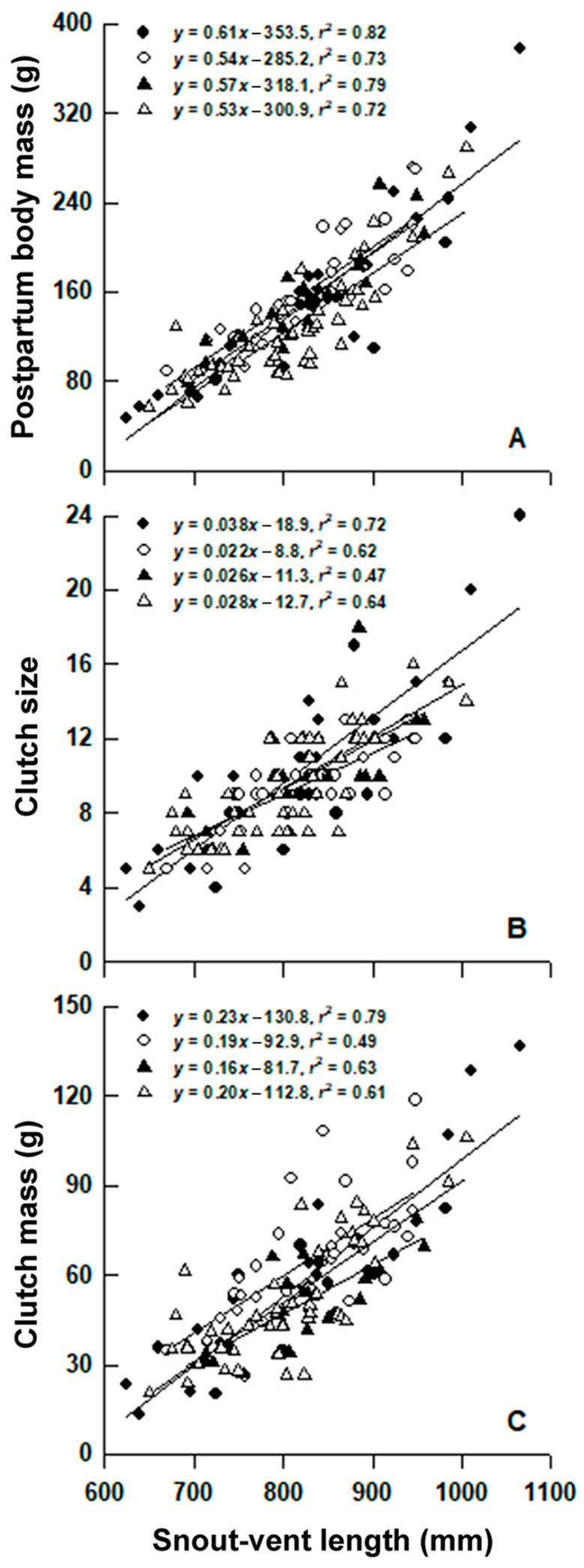
Postpartum body mass (**A**), clutch size (**B**), and clutch mass (**C**) in relation to female snout vent length. Regression equations and coefficients are given in the figure. ◆: 1999; ◊: 2010; ▲: 2011; △: 2014.

Postpartum body mass (Figure 3A), clutch size (Figure 3B), and clutch mass (Figure 3C) were positively related to female SVL in each year (linear regression analysis; all *p* < 0.001). Egg size was positively related to female SVL, but the trend was significant only in 1999 and 2014 (linear regression analysis; both *p* < 0.05). The proportions of variation in egg size were explained by maternal SVL varied from 5% (2010) to 15% (1999), far smaller than the values for postpartum body mass (72–82%; Figure 3A), clutch size (47–72%; Figure 3B), and clutch mass (49–79%; Figure 3C). Postpartum body mass, clutch mass, and egg size differed among years after accounting for female SVL, whereas clutch size did not (Table 1). More specifically, SVL-adjusted mean postpartum body mass was greater in 2010 than in 2014, SVL-adjusted mean clutch mass was greater in 2010 than in 2011 and 2014, and SVL-adjusted mean egg size was greater in 2010 than in the other three years (Table 1). Mean RCM was significantly lower in 2011 than in other three years (Table 1).

**Figure 4:**
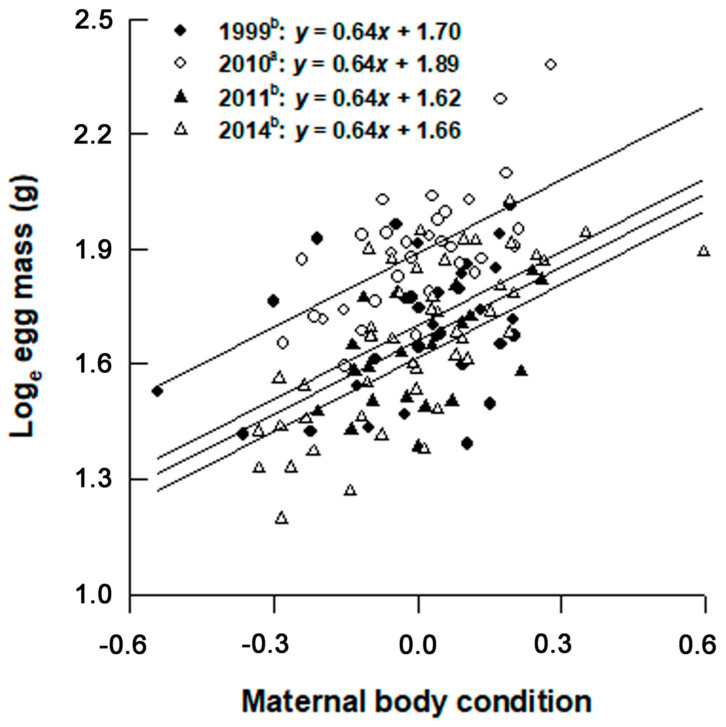
Egg mass in relation to postpartum body condition (calculated as regression residual of postpartum body mass against SVL). All data were log_e_ transformed. Regression lines were adjusted for the four years with a common slope (0.64) to facilitate comparisons. Years with different superscripts differ significantly (Tukey’s *post hoc* test, α = 0.05, a > b).

Within-population clutch size variability varied from 22% (2010) to 44% (1999), with a mean of 31%. In none of the four years was the correlation between clutch size and postpartum body mass significant after controlling for maternal SVL (partial correlation analysis; all *p* > 0.05). Within-population clutch mass variability varied from 30% (2011) to 49% (1999), with a mean of 38%. The (positive) correlation between clutch mass and postpartum body mass was significant only in 2011 after controlling for maternal SVL (partial correlation analysis; *r* = 0.33, *t* = 1.47, *df* = 18, *p* = 0.160). Within-population egg size variability varied from 14% (2011) to 21% (2014), with a mean of 17%. Within-clutch egg size variability was much lower (varying from 2.8% to 20.1%, with a mean of 5.7%) and displayed no annual variation (Table 1). The positive correlation between egg size and postpartum body mass was significant in each year after controlling for maternal SVL (partial correlation analysis; all *r* > 0.38 and all *p* < 0.05). Females did not trade off egg size against number, as revealed by the fact that the negative correlation between clutch mean egg mass and clutch size was not significant in each year after controlling for maternal SVL (partial correlation analysis; all *r* > −0.31 and all *p* > 0.05). Mean values for egg mass differed among years after accounting for maternal body condition (ANCOVA; *F*_3,126_ = 20.87, *p* < 0.0001). Setting maternal body condition at the average level of 0, we found that the mean egg mass was significantly greater in 2010 (6.7 g) than in other three years (5.1–5.5 g) (Figure 4).

## 4. Discussion

Mean maternal SVL, SVL-adjusted mean clutch size, and mean CV of egg mass (within-clutch egg size variability) showed no significant annual variation over the four years of our study, whereas mean and SVL-adjusted mean values for all other examined traits varied substantially among years (Table 1, Figure 2). As in other ectotherms with indeterminate growth, body size and age are highly correlated in snakes [33,34,35]. Therefore, the constancy in mean maternal SVL among years presumably suggests that the age or size structure of reproductive females remains consistent from year to year in the Zhejiang meta-population of *L. rufozonatus*. Previtellogenic body condition, a trait highly associated with food availability [36,37,38], is crucial to the onset of vitellogenesis, with females with a poor body condition often producing fewer offspring or even skipping current opportunities for reproduction [25,39]. Therefore, one plausible explanation for the constancy in SVL-adjusted mean clutch size among years lies in the fact that previtellogenic body conditions show little or even no annual variation in *L. rufozonatus*. Alternatively, it might be possible that previtellogenic conditions vary substantially over the years but are always better than the lower threshold required to initiate vitellogenesis of a clutch predicted by female SVL in *L. rufozonatus* [29]. The constancy in CV of egg mass among years suggests that female *L. rufozonatus* tend to produce offspring of a similar size within individual clutches. Such a trend in maternal investment per offspring more often occurs in fairly constant or predictable environments, fitting with the prediction from the parent–offspring conflict theory for maternal investment in individual offspring [5,40].

The timing of egg laying in squamate reptiles is determined by a complex interplay of abiotic and biotic factors [37,41,42,43]. Substantial variation in reproductive timing can occur even within a single population, and may generate important fitness variation [43,44,45,46]. In the present study, the inconsistency in egg laying date among years resulted, at least partly, from annual variation in seasonal weather conditions, which are of importance for reproduction because of their influence on habitat quality, food availability, foraging performance, and thus the acquisition of capital resources for reproduction [11,26,27,47]. Here, we found that females generally laid eggs earlier in 1999 and 2014 than in other two years (Figure 2). However, as the mean date was advanced by a maximum of ~5 days, the observed yearly changes in egg-laying date would be ecologically less important.

All other factors being equal, body mass should be greater in snakes with more energy reserves. Accordingly, the inconsistency in SVL-adjusted mean postpartum body mass among years suggests that, as in the water python *Liasis fuscus* [39] and the Arafura filesnake *Acrochordus arafurae* [48], energy reserves in postpartum females vary among years in *L. rufozonatus*. By contrast, SVL-adjusted mean postpartum body mass remains remarkably constant in the keelback *Tropidonophis mairii* [26], the king ratsnake *Elaphe carinata* [42], the Chinese cobra *Naja atra* [16], the pygmy rattlesnake *Sistrurus miliarius* [49], and the short-tailed pit viper *Gloydius brevicaudus* [50], where females tend to retain a set amount of energy reserves after laying. We found that SVL-adjusted mean clutch mass varied among years (Table 1). This variation resulted primarily from annual variation in egg size, because SVL-adjusted mean clutch size showed no annual variation (Table 1). RCM is determined by two variables, postpartum body mass and clutch mass, both of which may change temporally [32]. All else being equal, females producing heavier clutches but retaining a smaller amount of energy reserves at laying would display a higher RCM. Within a single population, annual variation in RCM may be apparent if food availability varies among years [51,52]. In the following, we focus our discussion on sources of variation in egg size, which is a key source of annual variation in reproductive output in *L. rufozonatus* (Table 1).

It is generalizable to snakes that total energy allocated to reproduction is positively related to maternal body size, but reproductive investment per offspring varies both within and among species [25]. Larger female *L. rufozonatus* also laid larger and heavier clutches (Figure 3), and they did so primarily by allocating available resources to produce more rather than larger eggs, as revealed by the fact that maternal SVL explained a small proportion (up to 15%) of variation in egg size. We did not detect the egg size number trade-off in *L. rufozonatum*. This affirms the results reported for three colubrid snakes of the genus *Ptyas*, *P. mucosus* [53], *P. korros* [54], and *P. dhumnades* [29], where females do not trade off egg size against number. By contrast, the egg size number trade-off is significant in the king ratsnake *Elaphe carinata* [42] and the Chinese cobra *Naja atra* [37] but, in both species, there is a fixed upper limit (~1.4 times greater that the population mean egg size) to egg size not set by the maternal body volume [15,16]. Our study is the first to demonstrate that postpartum body condition is an important source of variation in egg size in *L. rufozonatum*, as revealed by the fact that in each year, females with a better postpartum body condition produced larger eggs (Figure 4). Females laid larger eggs in 2010 than in other three years after accounting for maternal SVL (Table 1) or postpartum body condition (Figure 4). This finding is of particular interest because it suggests that factors other than maternal body size and postpartum body condition also affect egg size in *L. rufozonatus*. One of the most likely factors is current food availability. In many animals, energetic costs necessary for maintenance and growth should firstly be met, with reproduction having the lowest priority in energy allocation [55]. SVL-adjusted mean postpartum body mass was greater in 2010, suggesting that reproductive females accumulated a greater amount of energy before and/or during the breeding season in that year and were therefore better able to divert their current surplus energy to the production of larger eggs.

## 5. Conclusions

Larger females of *L. rufozonatus* produced larger and heavier clutches. This provides evidence for the traditional view that reproductive output (clutch or litter mass) is highly linked to maternal body size in snakes [25,46]. Of the two variables that determine reproductive output, egg size was more variable than clutch size after removing the influence of maternal body size. Thus, our study does not follow Smith and Fretwell’s (1974) [5] prediction that females with different amounts of resources to invest in reproduction should give priority to adjusting the number rather than size of their offspring. We found that females did not trade off egg size against number. This finding suggests that in *L. rufozonatus*, clutch size is not a significant source of variation in egg size. Egg size was positively related to maternal body size, although such a trend was significant only in 1999 and 2014; egg size was positively related to maternal postpartum body condition in each of the four years of our study. These findings suggest that maternal body size and body condition both are important sources of variation in egg size. Interestingly, females laid larger eggs in 2010 than in the other three years after accounting for their body size or body condition. This finding suggests that factors other than maternal body size and body condition may also affect the size of eggs produced by female *L. rufozonatus*.

## Figures and Tables

**Table 1:** Descriptive statistics, expressed as mean ± SE and range, for reproductive traits of female *L. rufozonatus* collected in 1999, 2010, 2011, and 2014, and results of one-way ANOVA [snout vent length (SVL) and coefficient of variation (CV) of egg mass] and ANCOVA (postpartum body mass, clutch size, clutch mass, and egg size with SVL as the covariate, and relative clutch mass with postpartum body mass as the covariate). Years with different superscripts differ significantly (Tukey’s *post hoc* test, α = 0.05, a > b).

	Year				ANOVA or ANCOVA Results
	1999	2010	2011	2014	
*N*	32	32	21	46	
Snout vent length (mm)	822.6 ± 19.2 625–1065	829.4 ± 13.4 670–948	819.0 ± 16.5 693–958	807.9 ± 12.0 650–1005	*F*_3,127_ = 0.42, *p* = 0.742
Postpartum body mass (g)	148.8 ± 12.9 47.3–378.2	161.6 ± 8.5 89.0–271.0	148.9 ± 10.5 78.0–256.5	128.1 ± 7.5 57.4–289.6	*F*_3,126_ = 4.19, *p* < 0.01 1999 ^ab^, 2010 ^a^, 2011 ^ab^, 2014 ^b^
Clutch size	10.4 ± 0.8 3–24	9.6 ± 0.4 5–13	9.8 ± 0.6 6–18	9.6 ± 0.4 5–16	*F*_3,126_ = 0.41, *p* = 0.734
Clutch mass (g)	58.1 ± 5.0 13.0–136.8	65.4 ± 3.7 26.1–118.6	50.3 ± 3.3 27.0–79.1	52.2 ± 3.1 20.6–106.1	*F*_3,126_ = 4.64, *p* < 0.01 1999 ^ab^, 2010 ^a^, 2011 ^b^, 2014 ^b^
Egg size (g)	5.5 ± 0.2 4.0–7.5	6.8 ± 0.2 4.9–10.8	5.1 ± 0.2 4.0–6.3	5.4 ± 0.2 3.3–7.6	*F*_3,126_ = 14.78, *p* < 0.0001 1999 ^b^, 2010 ^a^, 2011 ^b^, 2014 ^b^
CV of egg size (%)	6.8 ± 0.7 2.8–20.1	5.3 ± 0.5 1.7–12.8	5.0 ± 0.4 1.2–8.9	5.4 ± 0.3 2.1–11.0	*F*_3,127_ = 2.38, *p* = 0.073
Relative clutch mass	0.40 ± 0.02 0.23–0.64	0.41 ± 0.01 0.28–0.61	0.34 ± 0.01 0.22–0.47	0.40 ± 0.01 0.20–0.73	*F*_3,126_ = 5.29, *p* < 0.01 1999 ^a^, 2010 ^a^, 2011 ^b^, 2014 ^a^

## Data Availability

Not applicable.
